# Supplementary material for: Conformational interconversion of MLKL and disengagement from RIPK3 precede cell death by necroptosis
Source: Nat Commun. 2021 Apr 13;12:2211. doi: 10.1038/s41467-021-22400-z (PMC8044208; doi:10.1038/s41467-021-22400-z)
Supplement: Supplementary file 1 — Supplementary Information [file 41467_2021_22400_MOESM1_ESM.pdf]

SUPPLEMENTARY INFORMATION

Conformational interconversion of MLKL and disengagement from RIPK3 precede cell death by necroptosis

Sarah E. Garnish[1,2,8], Yanxiang Meng[1,2,8], Akiko Koide[3,4,8], Jarrod J. Sandow[1,2], Eric Denbaum[3], Annette V. Jacobsen[1,2], Wayland Yeung[5], Andre L. Samson[1,2], Christopher R. Horne[1,2], Cheree Fitzgibbon[1], Samuel N. Young[1], Phoebe P.C. Smith[1], Andrew I. Webb[1,2], Emma J. Petrie[1,2], Joanne M. Hildebrand[1,2], Natarajan Kannan[5,6], Peter E. Czabotar[1,2], Shohei Koide[3,7,9*], James M. Murphy[1,2,9*]

[1] Walter and Eliza Hall Institute of Medical Research, 1G Royal Parade, Parkville, VIC 3052, Australia
[2] Department of Medical Biology, University of Melbourne, Parkville, VIC 3052, Australia
[3] Perlmutter Cancer Center, New York University Langone Health, New York, NY 10016
[4] Department of Medicine, New York University Grossman School of Medicine, New York, NY 10016
[5] Institute of Bioinformatics, University of Georgia, Athens, GA, 30602, USA
[6] Department of Biochemistry and Molecular Biology, University of Georgia, Athens, GA 30602, USA
[7] Department of Biochemistry and Molecular Pharmacology, New York University Grossman School of Medicine, New York, NY 10016

[8] These authors contributed equally: Sarah E. Garnish, Yanxiang Meng, Akiko Koide
[9] These authors jointly supervised this work: Shohei Koide, James M. Murphy
[*]To whom correspondence should be addressed: Shohei.Koide@nyulangone.org or jamesm@wehi.edu.au

SUPPLEMENTARY FIGURES

Supplementary Figure 1. Sequences of the human MLKL pseudokinase domain-binding Monobodies, Mb26, Mb27 and Mb32. a Monobody library designs and sequences of Mb32, Mb26 and Mb27 (adapted from ref.[1]; Mb32 sequence reported previously[2]). Varied positions in the "side and loop" and "loop only" Monobody libraries are shown in red. "X" denotes a mixture of 30% Tyr, 15% Ser, 10% Gly, 5% Phe, 5% Trp and 2.5% each of all other amino acids (except Cys); "B" denotes a mixture of Gly, Ser, Tyr; "J", Ser and Tyr; "O" a mixture of Asp, Asn, His, Leu, Ile, Val, Phe and Tyr; "U", a mixture of His, Leu, Phe and Tyr; "Z", a mixture of Ala, Lys, Glu and Thr. **b-c** Binding titration of Mb26 and Mb27 for recombinant human MLKL pseudokinase domain measured by yeast display. The K_D value is shown ±S.D.; data are plotted as mean ± S.D for triplicate measurements. The K_D of Mb32 for human MLKL pseudokinase domain is 37.1 ± 2.4 nM, and was previously reported[2].

Supplementary Figure 1

a

Clone	K_D (nM)	Amino acid sequence
		10 20 **BC** 30 40 50 **DE** 60 70 **FG** 90
library		VSSVPTKLEVVAATPTSLLISWDA (X_{4-8}) VXYYRITYGETGGNSPVQEFTVPBJJJTATISGLSPGVDYTITVYA (X_{7-13}) SPISINYRT
Mb(MLKL_26)	176±13	VSSVPTKLEVVAATPTSLLISWDAEYEMGWWDVRYYRITYGETGGNSPVQEFTVPGSSSTATISGLSPGVDYTITVYANYYSGYSIEEYSPISINYRT
Mb(MLKL_27)	75±14	VSSVPTKLEVVAATPTSLLISWDAPYTYYW--VDYYRITYGETGGNSPVQEFTVPGSSSTATISGLSPGVDYTITVYAYDYGGWWAY--SPISINYRT
		βA βB βC βD βE βF βG

Clone	K_D (nM)	Amino acid sequence
		10 20 **βC** 40 **CD** **βD** 50 60 70 **FG** 90
library		VSSVPTKLEVVAATPTSLLISWDAPAVTVOUYOITYGETGGNSPVQZFZVPGSKSTATISGLSPGVDYTITVYA (X_{7-13}) SPISINYRT
Mb(MLKL_32)	37.1±2.4	VSSVPTKLEVVAATPTSLLISWDAPAVTVDLYIITYGETGGNSPVQTFEVPGSKSTATISGLSPGVDYTITVYAYSFMYHDYYYPEWSPISINYRT
		βA βB βC βD βE βF βG

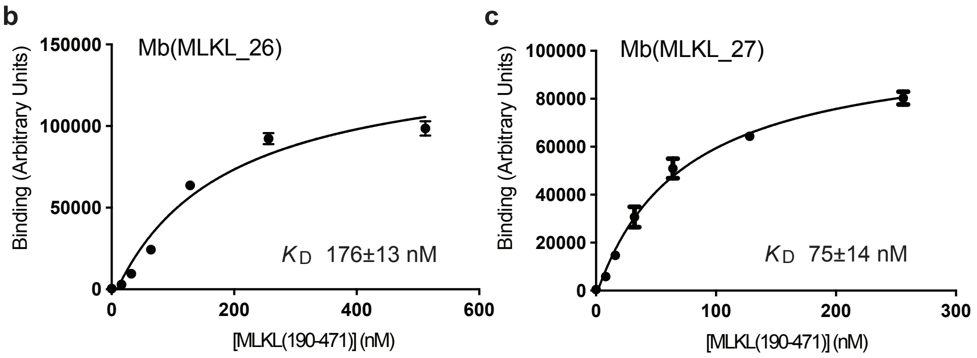

Supplementary Figure 2. Binding of Mb26, Mb27 or Mb32 to MLKL does not inhibit necroptosis. **a** Detection of FLAG-Mb26-GFP, FLAG-Mb27-GFP and FLAG-Mb32-GFP in wild-type (WT) HT29 cells following doxycycline (dox) induction via FLAG western blot. **b-d** The effect of dox-induced Mb26, Mb27 and Mb32 expression on WT HT29 cells was evaluated in untreated (UT) and necroptotic (TSI; TNF, Smac mimetic Compound A, pan-caspase inhibitor IDN-6556) conditions. Cell death was measured by propidium iodide (PI) uptake and flow cytometry; death data represent mean ± SEM. One independent cell line was generated for each Monobody; the Mb26 line was assayed in n=3 independent experiments, whilst the Mb27 and Mb32 lines were assayed in n=4 independent experiments. **e** Mb26 immunoprecipitated MLKL from lysates of wild-type HT29 cells only following necroptotic stimulation (TSI, 3 h). **f** Detection of Mb26 and Mb27 in *RIPK3*$^{-/-}$ HT29 cells via FLAG western blot. **g** FLAG-IP of Mb26 in *RIPK3*$^{-/-}$ HT29 cells stimulated with and without overnight dox-induction and TSI necroptotic stimulation. **h** Detection of Mb32 in *MLKL*$^{-/-}$ HT29 cells via FLAG western blot. **i** FLAG-IP of Mb32 in *MLKL*$^{-/-}$ HT29 cells stimulated with and without overnight doxycycline induction. A representative of independent duplicates is shown in panels a-i. **j** Wild-type (WT) and Q356A human MLKL expression was induced with doxycycline and cell death was measured by PI uptake using flow cytometry in the presence or absence of 24 h TSI stimulation. WT and Q356A exogenes were expressed in 3 and 2 independent *MLKL*$^{-/-}$ U937 clones, respectively. Two independent WT lines and one Q356A line were assayed in n=2 experiments, with the remaining lines assayed in n=1 experiment. Death data shown as mean ± SEM. **k** Wild-type (WT) and E250A human MLKL expression was induced in *MLKL*$^{-/-}$ HT29 cells with dox and cell death was measured by SYTOX Green uptake (1/mm^2) quantified using IncuCyte S3 live cell imaging in the presence or absence of a necroptotic stimulus (TSI) for 24 h. One independent cell line was generated for each of WT and E250A human MLKL; each line was assayed in n=4 independent experiments. Data are plotted as mean ± SEM. **l, m** A representation of the flow cytometry gating strategy used to collect the data in panels b-d and j. Initially, a gate is applied to exclude cellular debris (l) before analysis of PI-positivity as a measure of cell death (m) is performed.

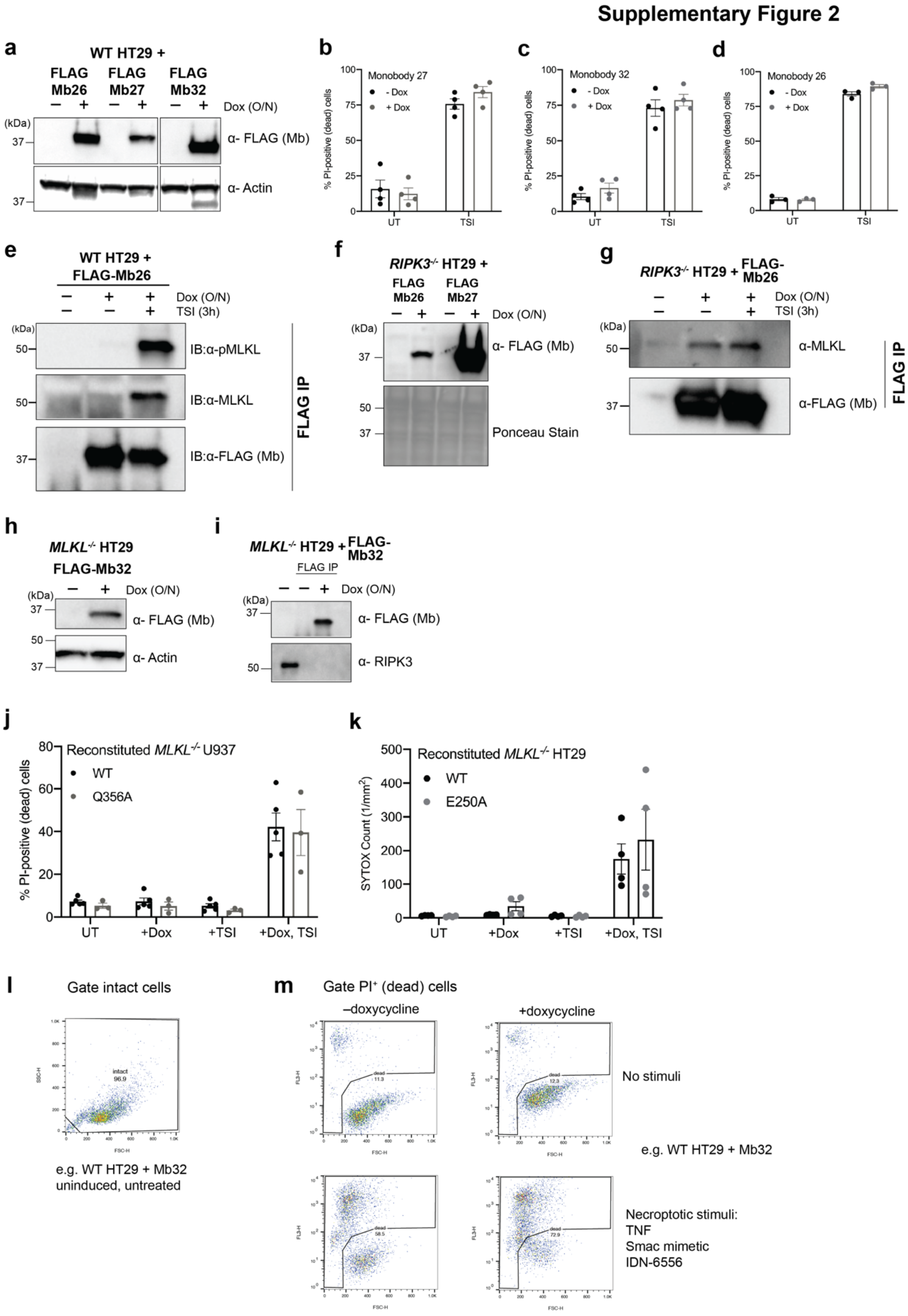

Supplementary Figure 2
a
WT HT29 +
FLAG Mb26
FLAG Mb27
FLAG Mb32
Dox (O/N)
(kDa)
37
α- FLAG (Mb)
α- Actin
b
Monobody 27
- Dox
+ Dox
% PI-positive (dead) cells
UT
TSI
c
Monobody 32
d
Monobody 26
e
WT HT29 +
FLAG-Mb26
Dox (O/N)
TSI (3h)
IB:α-pMLKL
IB:α-MLKL
IB:α-FLAG (Mb)
FLAG IP
f
RIPK3-/- HT29 +
FLAG Mb26
FLAG Mb27
α- FLAG (Mb)
Ponceau Stain
g
RIPK3-/- HT29 + FLAG-Mb26
α-MLKL
α-FLAG (Mb)
FLAG IP
h
MLKL-/- HT29
FLAG-Mb32
α- FLAG (Mb)
α- Actin
i
MLKL-/- HT29 + FLAG-Mb32
FLAG IP
α- FLAG (Mb)
α- RIPK3
j
Reconstituted MLKL-/- U937
WT
Q356A
% PI-positive (dead) cells
UT
+Dox
+TSI
+Dox, TSI
k
Reconstituted MLKL-/- HT29
WT
E250A
SYTOX Count (1/mm2)
l
Gate intact cells
e.g. WT HT29 + Mb32
uninduced, untreated
m
Gate PI+ (dead) cells
–doxycycline
+doxycycline
No stimuli
e.g. WT HT29 + Mb32
Necroptotic stimuli:
TNF
Smac mimetic
IDN-6556

Supplementary Figure 3. Mb26, Mb27 and Mb32 bind human MLKL, but only Mb32 binds MLKL:RIPK3 complexes. a-e His_6-tagged recombinant Monobodies were mixed with recombinant MLKL pseudokinase domain (MLKL) (**a**) or recombinant MLKL pseudokinase domain-RIPK3 kinase domain heterocomplex (MLKL:RIPK3) (**b-e**) and loaded onto a HisTrap HP column in buffer supplemented with 5 mM imidazole. Bound proteins were eluted in buffer supplemented with 300 mM imidazole and each fraction was analysed by reducing SDS-PAGE with Stain-free imaging (TGX Stain-free gels, Biorad). Mb32 co-eluted with MLKL (**a**) and MLKL:RIPK3 (**b**). Mb26 (**c**) and Mb27 (**d**) co-eluted with dissociated MLKL, but not the MLKL:RIPK3 complex. **e** As expected, Mb33, an N-terminal four-helix bundle domain ligand, did not bind MLKL:RIPK3. Each shown experiment is representative of two independent repeats.

Supplementary Figure 3

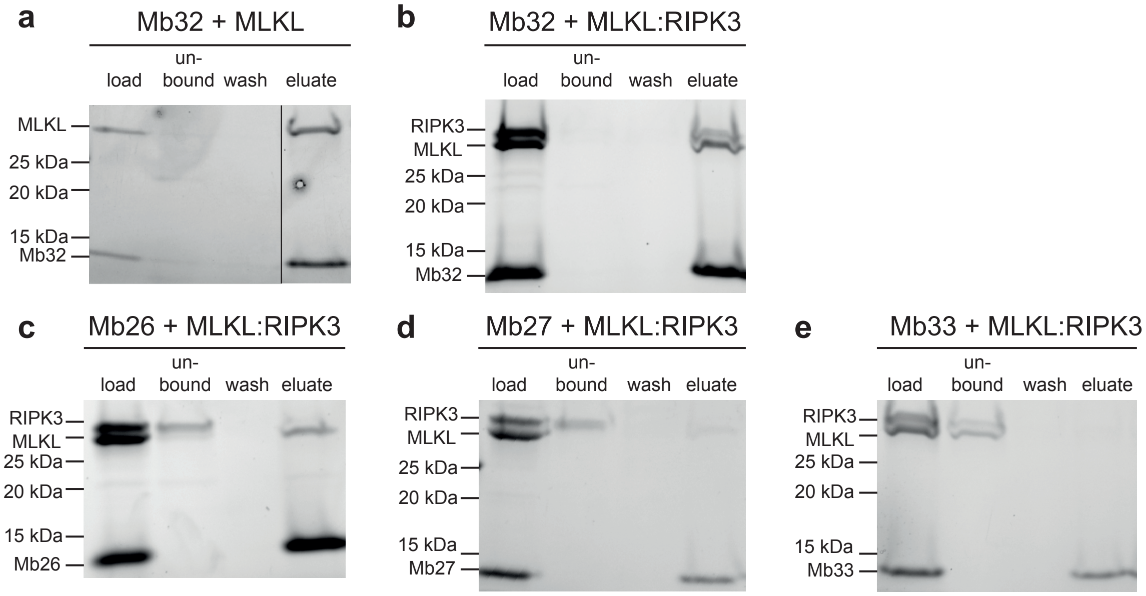

Supplementary Figure 4. Mass spectrometry validation of Mb27 immunoprecipitation of endogenous human MLKL after necroptotic stimulation. HT29 cells were treated with doxycycline to induce expression of FLAG-tagged Mb27 or Mb32 before 3h stimulation with TSI and subsequent cell lysis. Monobodies and interacting partners were enriched through FLAG-tag immunoprecipitation. Signed P value ($-\log_{10}$ P value for each comparison assigned a positive or negative value based on $\log_2$ fold change) volcano plots of protein enrichment in cells (with background proteins from untreated (UT) cells subtracted) stimulated with TSI (y-axis) compared with doxycycline (Dox) only (x-axis) for (**a**) Mb27 and (**b**) Mb32. **a** MLKL was the most significant hit in Mb27 immunoprecipitates from TSI-stimulated (y-axis), but not unstimulated (x-axis) HT29 cells. **b** MLKL was the most significant hit in Mb32 immunoprecipitates from untreated (UT; x-axis) and necroptosis stimulated (TSI; y-axis) HT29 cells. The top 20 proteins enriched by each Monobody are labelled; MLKL highlighted in red and known necrosomal proteins highlighted in bold text. P values calculated using the Limma (v3.40.6) R (v3.6.1) package (data derived from n=4 independent immunoprecipitation experiments).

Supplementary Figure 4

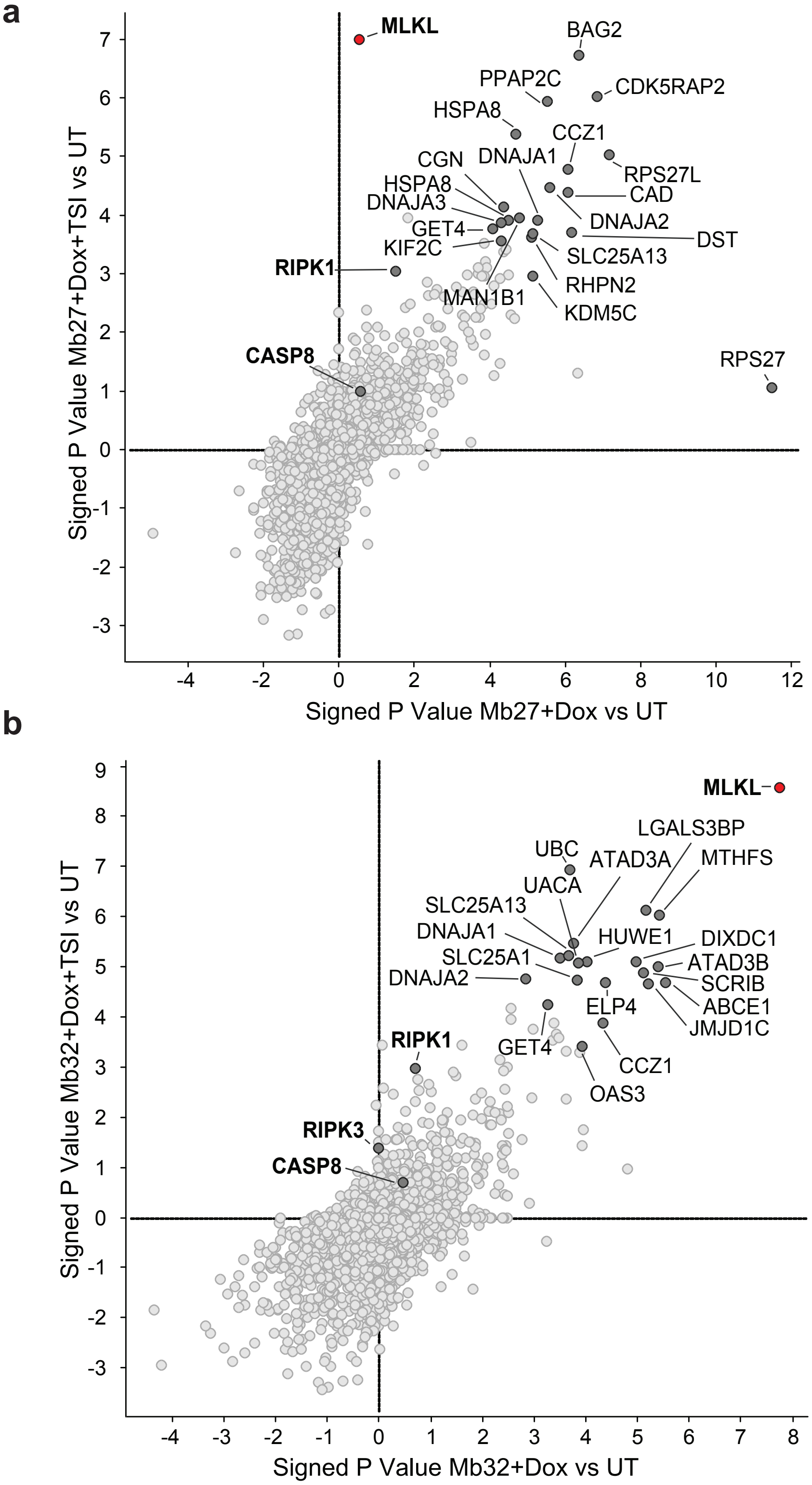

Supplementary Figure 5. Electron density of K230-mediated interactions in each MLKL structure; the open MLKL conformation is incompatible with Mb27 binding. a The salt bridge between K230 and E250 in the Mb27-bound MLKL pseudokinase domain. **b** The Mb27 (blue; teal):MLKL (grey) complex was superimposed with the Mb32-bound conformation of human MLKL pseudokinase domain (grey; activation loop, green; αC helix, yellow), revealing that clashes would occur between Mb27 and the activation loop of the open form of MLKL. **c** The salt bridge between K230 and E250 is displaced by the activation loop helix, where Q356 forms a hydrogen bond with K230. Structural models in **a** and **c** are shown as sticks; the 2Fo-Fc maps (grey mesh) are contoured at 1σ.

Supplementary Figure 5

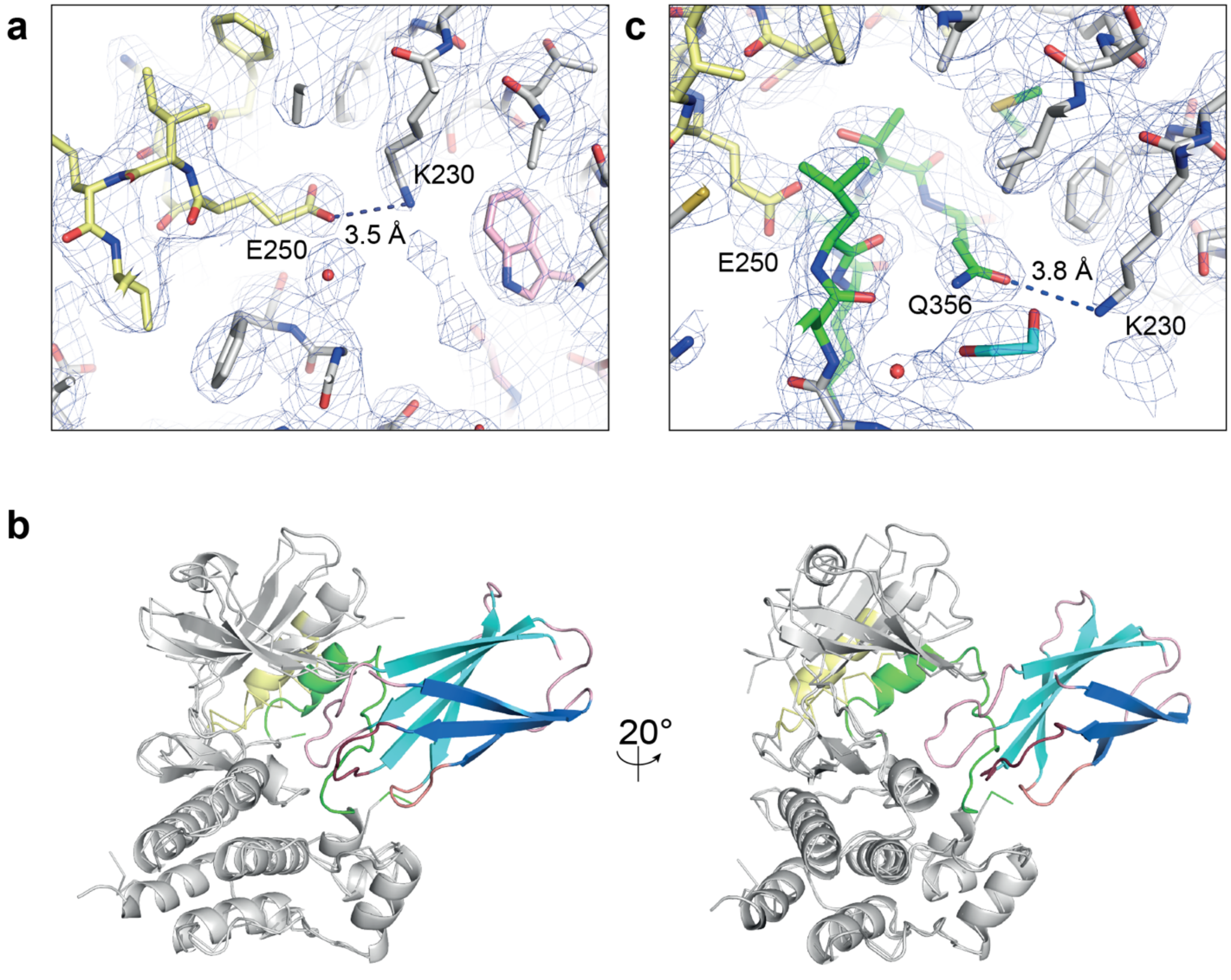

Supplementary Figure 6. Mutation of human MLKL R292 or T374 within the Mb27 epitope perturbs the kinetics of necroptosis. a *MLKL*$^{-/-}$ HT29 cells were stimulated with doxycycline (dox) to induce expression of wild-type (WT) and mutant human MLKL constructs, and protein levels were detected by immunoblotting. Data are representative of duplicate independent experiments. **b-u** The effects of alanine substitutions within the Mb27-binding epitope of human MLKL on the kinetics of necroptosis were assessed in *MLKL*$^{-/-}$ HT29. Wild-type or mutant human MLKL expression was induced with dox and cell death was measured by SYTOX Green uptake (1/mm^2) quantified using IncuCyte S3 in the presence or absence of a necroptotic stimulus (TSI) every hour for 20 h. Two independent cell lines were generated for WT, S239A, Q236A, and one for all other MLKL mutants. WT lines were each assayed in n=5 independent experiments; TSEE, R333A and one independent Q236A line were assayed in n=4 independent experiments; all other mutants were assayed in n=3 independent experiments. Data are plotted as mean ± SEM.

Supplementary Figure 6

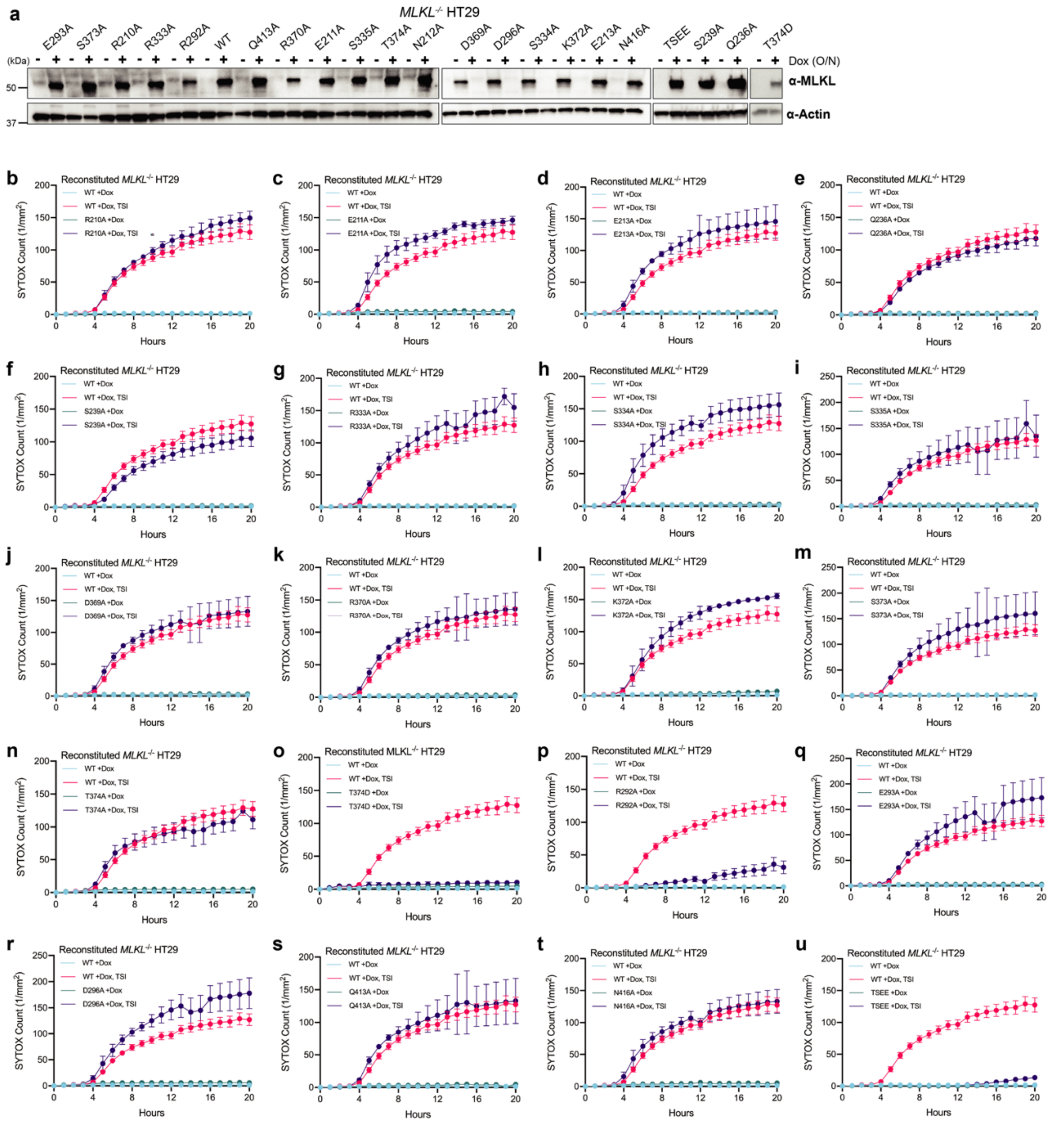

Supplementary Figure 7. Mutation of T374 within the Mb27 epitope perturbs the kinetics of phosphatidylserine exposure. The effects of alanine substitutions within the Mb27-binding epitope of human MLKL on the kinetics of phosphatidylserine exposure were assessed in *MLKL*$^{-/-}$ HT29. Wild-type or mutant human MLKL expression was induced with doxycycline (Dox) and cell death phosphatidylserine exposure as readout by Annexin V-positivity (1/mm^2) was quantified using IncuCyte S3 in the presence or absence of a necroptotic stimulus (TSI) at 20 h. Two independent cell lines were generated for WT, S239A, Q236A, and one for all other MLKL mutants. One WT line was assayed in n=4 independent experiments; S239A, Q236A, and T374D independent lines were assayed in n=2 independent experiments and all other mutants were assayed in n=3 independent experiments. Data are plotted as mean ± SEM. **b** Mb27 immunoprecipitates wild-type, but not R292A or T374D, human MLKL expressed in *MLKL*$^{-/-}$ HT29 cells. Data are representative of duplicate independent experiments.

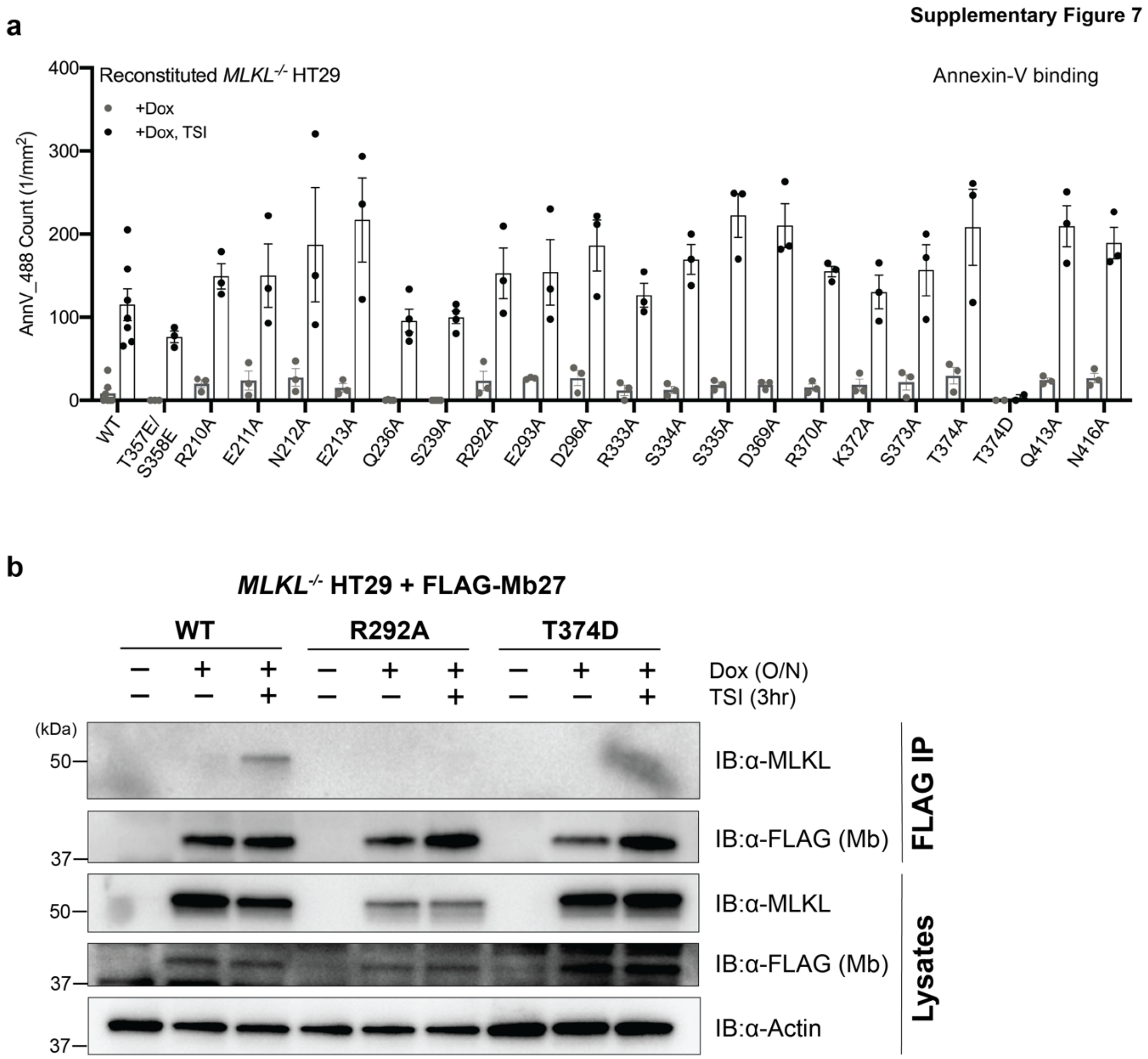

SUPPLEMENTARY TABLE

Supplementary Table 1 | Oligonucleotide sequences

hRIPK3 316 stop KpnI rev‡	5´- CTCCCCCATCTCCCGTTAagaaaatctcctattgctgctcctgag-3´
NcoI RBS hRIPK3 C3S fwd	5´- GGATCTCGAGCCATGGAAACCATGtcgTCCgtcaagttatggccc-3´
pFB_HTb AccI fwd	5´-AGCTATAGTTCTAGTGGTTGGCTACGT-3´
pFB_HTb AccI rev	5´-AGAGTCCTGGGCGAACAAACG-3´
pAB2 AccI polyA fwd	5´-TTCGCCCAGGACTCTCCCCGCGTTTATGAACAAACG-3´
pAB2 p10 rev	5´-ACTAGAACTATAGCTCGGACCTTTAATTCAACCCAACACAA-3´
Monobody BamHI fwd	5´- CGCGGATCCGTTTCTTCTGTTCCGACCAAAC-3´
Monobody no stop NheI rev	5´- TTCGCTGTGCTAGCGGTACGGTAGTTAATCGAGATTG-3´
Monobody stop NheI rev	5´- TTCGCTGTGCTAGCtaGGTACGGTAGTTAATCGAGATTG-3´
Monobody no stop XbaI rev	5´- agttactagTCTAGAggtacggtagttaatcgagattg-3´
Monobody stop XbaI rev	5´- agttactagTCTAGATTAggtacggtagttaatcgagattg-3´
Monobody no stop NheI rev	5´- TTCGCTGTGCTAGCGGTACGGTAGTTAATCGAGATTG-3´
hMLKL Q236A fwd	5´- gtattcaaaaaactcGCggctggcagc-3´
hMLKL Q236A rev	5´- gctgccagccGCgagtttttgaatac-3´
hMLKL S239A fwd	5´- ctccaggctggcGCcattgcaatagtgag-3´
hMLKL S239A rev	5´- ctcactattgcaatgGCgccagcctggag-3´
hRIPK3 exon 2 sgRNA	5´- GAATTCGTGCTGCGCCTAGA -3´
hMLKL 190 BamHI fwd	5´- CGCGGATCCcaagagcaaatcaaggagatcaag-3´
hMLKL 471 no stop NheI rev	5´- TTTCGCTGTGCTAGCcttagaaaaggtggagagtttc-3´
hMLKL 471 stop EcoRI rev	5´- CGCGAATTcacttagaaaaggtggagagtttc-3´

‡ Restriction sites underlined

SUPPLEMENTARY REFERENCES

1. Koide A, Wojcik J, Gilbreth RN, Hoey RJ, Koide S. Teaching an old scaffold new tricks: monobodies constructed using alternative surfaces of the FN3 scaffold. *J Mol Biol* **415**, 393-405 (2012).

2. Petrie EJ*, et al.* Identification of MLKL membrane translocation as a checkpoint in necroptotic cell death using Monobodies. *Proc Natl Acad Sci U S A* **117**, 8468-8475 (2020).
