# Supplementary material for: Conformational interconversion of MLKL and disengagement from RIPK3 precede cell death by necroptosis
Source: Nat Commun. 2021 Apr 13;12:2211. doi: 10.1038/s41467-021-22400-z (PMC8044208; doi:10.1038/s41467-021-22400-z)
Supplement: Supplementary file 3 — Reporting Summary [file 41467_2021_22400_MOESM3_ESM.pdf]

nature research

Corresponding author(s): Shohei Koide
James M. Murphy

Last updated by author(s): Mar 4, 2021

Reporting Summary

Nature Research wishes to improve the reproducibility of the work that we publish. This form provides structure for consistency and transparency in reporting. For further information on Nature Research policies, see our Editorial Policies and the Editorial Policy Checklist.

Statistics

For all statistical analyses, confirm that the following items are present in the figure legend, table legend, main text, or Methods section.

n/a	Confirmed	
☐	☒	The exact sample size (n) for each experimental group/condition, given as a discrete number and unit of measurement
☐	☒	A statement on whether measurements were taken from distinct samples or whether the same sample was measured repeatedly
☒	☐	The statistical test(s) used AND whether they are one- or two-sided<br>*Only common tests should be described solely by name; describe more complex techniques in the Methods section.*
☒	☐	A description of all covariates tested
☒	☐	A description of any assumptions or corrections, such as tests of normality and adjustment for multiple comparisons
☒	☐	A full description of the statistical parameters including central tendency (e.g. means) or other basic estimates (e.g. regression coefficient) AND variation (e.g. standard deviation) or associated estimates of uncertainty (e.g. confidence intervals)
☒	☐	For null hypothesis testing, the test statistic (e.g. F, t, r) with confidence intervals, effect sizes, degrees of freedom and P value noted<br>*Give P values as exact values whenever suitable.*
☒	☐	For Bayesian analysis, information on the choice of priors and Markov chain Monte Carlo settings
☒	☐	For hierarchical and complex designs, identification of the appropriate level for tests and full reporting of outcomes
☒	☐	Estimates of effect sizes (e.g. Cohen's d, Pearson's r), indicating how they were calculated

Our web collection on statistics for biologists contains articles on many of the points above.

Software and code

Policy information about availability of computer code

Data collection	Crystallographic data were collected using MX controls (Australian Synchrotron MX beamline control software; in-house, no versioning).<br>GROMACS 2016.4 was used to perform molecular dynamics simulations with CHARMM36 force field structure parameterization. Molecular dynamics umbrella samples used the Weighted Histogram Analysis Method (WHAM v2.0.10). Incucyte S3 data were collected on an instrument with firmware version 20202.2.0.0.
Data analysis	XDS (version March 15, 2019), AIMLESS (CCP4i2 Suite version 7.0.078) , PHASER (version 2.8.3), PHENIX (version 1.18.2-3874), COOT (version 0.9) and MOLPROBITY (Phenix Suite version 1.18.2-3874) were used for X-ray crystal structure determination.<br>PDBePISA (version 1.52) was used to analyze the protein oligomeric interface.<br>PyMOL (version 2.3.4) was used for structure analysis and cartoon depictions.<br>Incucyte S3 data were analysed user the controller software version 2020B Rev1.<br>R version 3.6.1 and Limma version 3.40.6 were used for mass spectra analyses.

For manuscripts utilizing custom algorithms or software that are central to the research but not yet described in published literature, software must be made available to editors and reviewers. We strongly encourage code deposition in a community repository (e.g. GitHub). See the Nature Research guidelines for submitting code & software for further information.

Data

Policy information about availability of data

All manuscripts must include a data availability statement. This statement should provide the following information, where applicable:

- Accession codes, unique identifiers, or web links for publicly available datasets
- A list of figures that have associated raw data
- A description of any restrictions on data availability

The atomic coordinates for the human MLKL pseudokinase domain complexes with Mb27 and Mb32 have been deposited in the Protein Data Bank with the accession numbers, 7JW7 [http://doi.org/10.2210/pdb7JW7/pdb] and 7JXU [http://doi.org/10.2210/pdb7JXU/pdb], respectively.
Source data are provided for Figures 1, 4g, Supplementary Figures 2a, 2e-i, 6a and 7b (immunoblots); Figure 4e and Supplementary Figures 2k, 6b-u, 7a (IncuCyte S3 data); and Supplementary Figure 2b-d, j (flow cytometry).
Other data, including expression construct sequences, are available from the corresponding authors upon request.

Field-specific reporting

Please select the one below that is the best fit for your research. If you are not sure, read the appropriate sections before making your selection.

[x] Life sciences [ ] Behavioural & social sciences [ ] Ecological, evolutionary & environmental sciences

For a reference copy of the document with all sections, see nature.com/documents/nr-reporting-summary-flat.pdf

Life sciences study design

All studies must disclose on these points even when the disclosure is negative.

Sample size	All cell-based experiments were performed typically with singlicate human cell lines, as noted in methods and legends. The numbers of independent repeat experiments are stated in figure legends. Because our prior studies have shown robust consistency between assays, n of 3 or more were established as sufficient to reveal any differences between cell lines.
Data exclusions	Some death assay repeats were excluded from analysis where there was suspected technical error i.e. high levels of cell death in uninduced untreated cell lines. These data were excluded in entirety from analysis in these circumstances.
Replication	Experiments were repeated independently typical at least in triplicate, as stated in legends, except some experiments that were performed in duplicate as stated. In cases where there was variability between repeats more repeats were generated. With the exception of obvious technical error as stated above, all repeats were used in analysis. The natural spread of the data is shown in the paper through use of individual data points and standard error of the mean error bars. All attempts at replication were successful, except on rare occasions where technical errors, such as omission of dox to induce gene expression or TSI to induce death, occurred.
Randomization	This is not relevant to our study. Our study compared the death of cell lines expressing different exogenes upon stimulation in parallel, or the binding activities of different purified proteins in parallel.
Blinding	This is not relevant to our study. Our study compared the death of cell lines expressing different exogenes upon stimulation in parallel, or the binding activities of different purified proteins in parallel.

Reporting for specific materials, systems and methods

We require information from authors about some types of materials, experimental systems and methods used in many studies. Here, indicate whether each material, system or method listed is relevant to your study. If you are not sure if a list item applies to your research, read the appropriate section before selecting a response.

Materials & experimental systems

n/a	Involved in the study	
[ ]	[x]	Antibodies
[ ]	[x]	Eukaryotic cell lines
[x]	[ ]	Palaeontology and archaeology
[x]	[ ]	Animals and other organisms
[x]	[ ]	Human research participants
[x]	[ ]	Clinical data
[x]	[ ]	Dual use research of concern

Methods

n/a	Involved in the study	
[x]	[ ]	ChIP-seq
[ ]	[x]	Flow cytometry
[x]	[ ]	MRI-based neuroimaging

Antibodies

Antibodies used	Primary antibodies used in this study were: rat anti-MLKL (clone 3H1, produced in-house; 1:1000 dilution; available as MABC604,

	EMD Millipore, Billerica, MA, USA), rat anti-human MLKL pseudokinase domain (clone 7G2, produced in-house; 1:2000 dilution), rabbit anti- human MLKL phospho-S358 (AB187091, Abcam; 1:3000), mouse anti-Actin (A-1978, Sigma-Aldrich, St Louis, MO, USA; 1:5000), rat anti-human RIPK3 (clone 1H2, produced in-house; 1:1000), rabbit anti- human phospho-S227 (D6W2T, CST, 1:1000), mouse anti-FLAG M2 (Sigma- Aldrich, F1804, 1:3000).
Validation	MLKL, pMLKL, RIPK3, pRIPK3 antibodies have been validated for selectivity using KO cell lines in Samson et al. Cell Death Differ (2021). Sigma anti-FLAG has been validated by the manufacturer (https://www.sigmaaldrich.com/catalog/product/sigma/f1804), and also in our study where no signal was observed prior to induction of exogenous FLAG-tagged Monobodies. Anti-Actin has been validated by the manufacturer (https://www.sigmaaldrich.com/catalog/product/sigma/a1978).

Eukaryotic cell lines

Policy information about cell lines

Cell line source(s)	MLKL-deficient U937 cells are as described in Petrie et al. Nat Comm 2018 and are derived from parental cells supplied by ATCC. HEK293T cells used for lentivirus generation were sourced from ATCC. HT29 cells were supplied by Mark Hampton an originate from ATCC. CRISPR-edited derivative HT29 cells lacking MLKL were reported previously (Petrie et al., Nature Commun 2018) while RIPK3-deficient HT29 cells are described for the first time in the present study. Sf21 cells were used for insect cell expression (purchased from Thermo).
Authentication	None of the cells were formally authenticated. However, their morphologies are consistent with the stated cell type. The lack of responsiveness of MLKL- or RIPK3-deficient human HT29 cells to necroptotic stimuli and the lack of respective MLKL or RIPK3 reactivity by western blot are consistent with the absence of MLKL or RIPK3. Sf21 cells were susceptible to baculovirus infection and produced recombinant proteins encoded by these baculoviruses, consistent with their authenticity.
Mycoplasma contamination	All mammalian lines used were monitored for mycoplasma and were found to be negative in our routine screening.
Commonly misidentified lines (See ICLAC register)	Nil

Flow Cytometry

Plots

Confirm that:

- [x] The axis labels state the marker and fluorochrome used (e.g. CD4-FITC).
- [x] The axis scales are clearly visible. Include numbers along axes only for bottom left plot of group (a 'group' is an analysis of identical markers).
- [x] All plots are contour plots with outliers or pseudocolor plots.
- [x] A numerical value for number of cells or percentage (with statistics) is provided.

Methodology

Sample preparation	Cells were stained with propidium iodide as a marker of death. This is a very simple, routine and robust assay, which does not warrant presentation of primary plots. For this reason, the above plot information is not relevant. Flow is used simply to enumerate the number of dead (PI+) cells.
Instrument	BD FACSCalibur
Software	FlowJo 10.1r7
Cell population abundance	Data were collected until 5000 events were recorded in the intact cell gate. Flow cytometry was used solely to quantify the number of dead (PI+) cells in a population for some cell death assays shown in Supplementary Figures
Gating strategy	We have previously reported the gating strategy (Murphy et al. Immunity 2013 and Davies et al. Nature Commun 2020). We have provided an example gating strategy as Supp. Fig. 2l-m. Briefly cells are first gated to include only intact cells, based on forward and side scattering, then PI positive cells are gated as the 'dead' population.

- [x] Tick this box to confirm that a figure exemplifying the gating strategy is provided in the Supplementary Information.
