# Supplementary material for: Conformational interconversion of MLKL and disengagement from RIPK3 precede cell death by necroptosis
Source: Nat Commun. 2021 Apr 13;12:2211. doi: 10.1038/s41467-021-22400-z (PMC8044208; doi:10.1038/s41467-021-22400-z)

Figure 1A

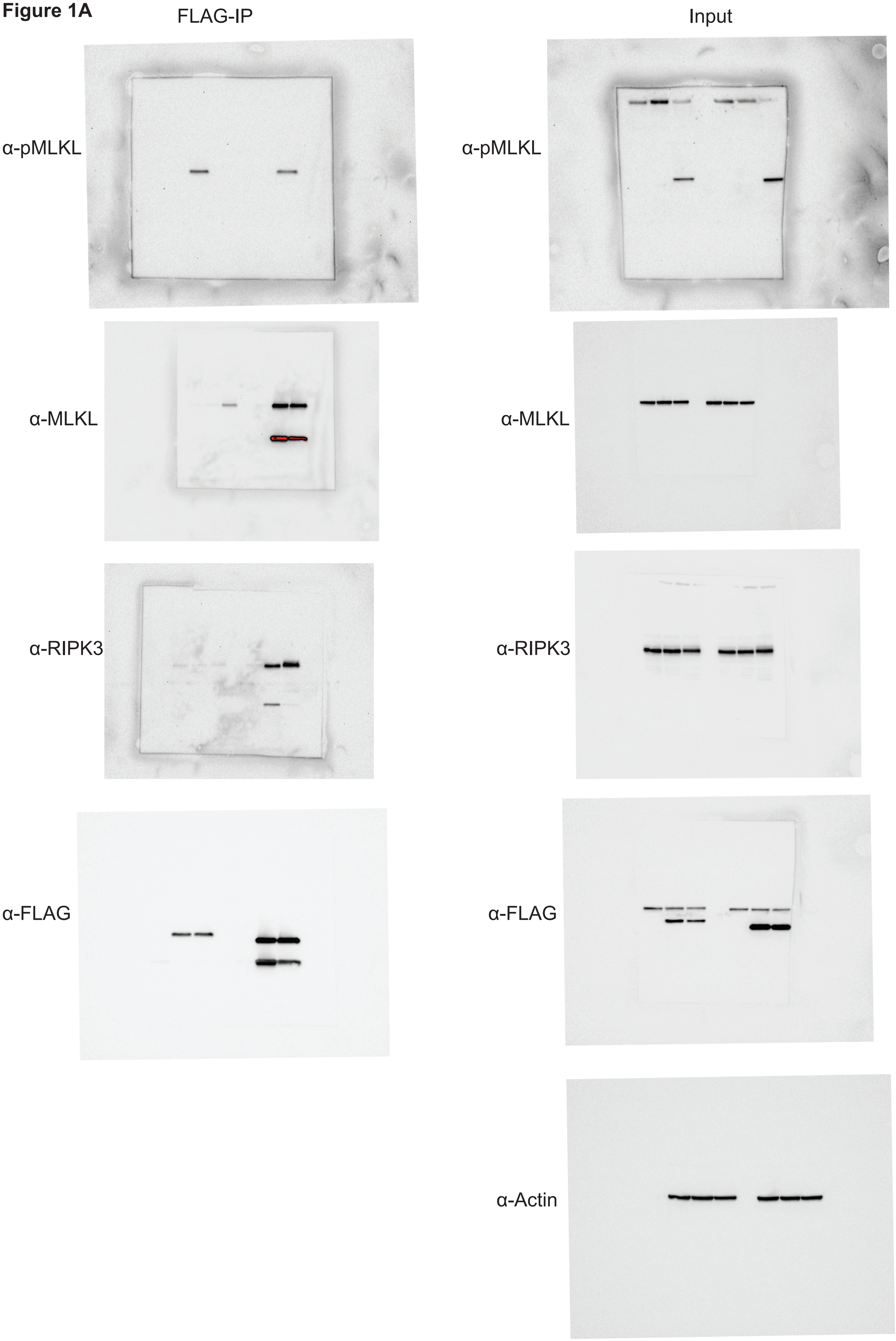
FLAG-IP
Input
α-pMLKL
α-pMLKL
α-MLKL
α-MLKL
α-RIPK3
α-RIPK3
α-FLAG
α-FLAG
α-Actin

Figure 1B

α-MLKL

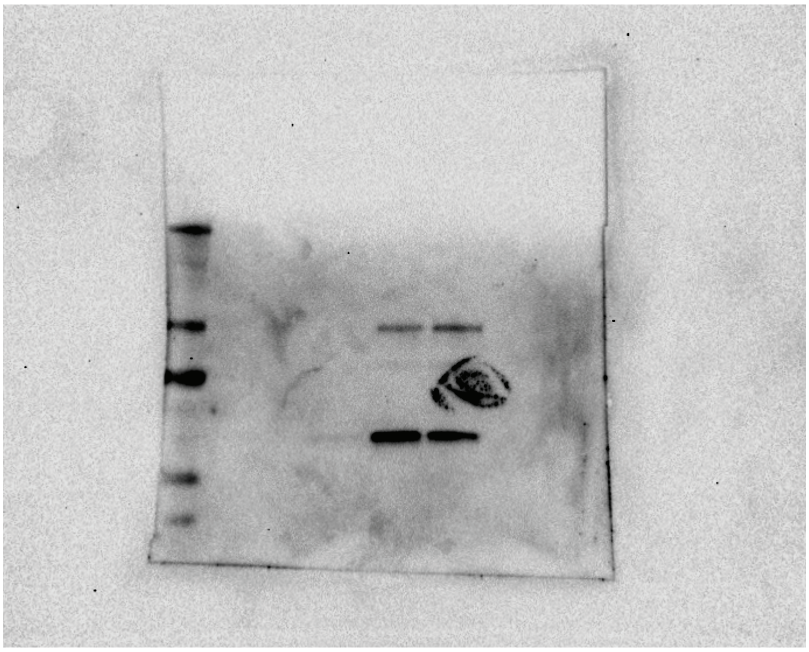

α-FLAG

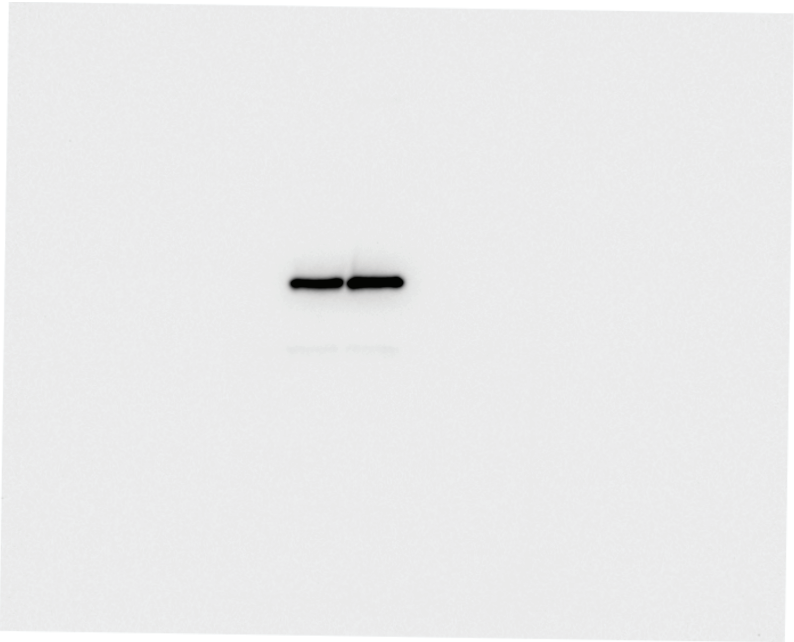

α-pMLKL

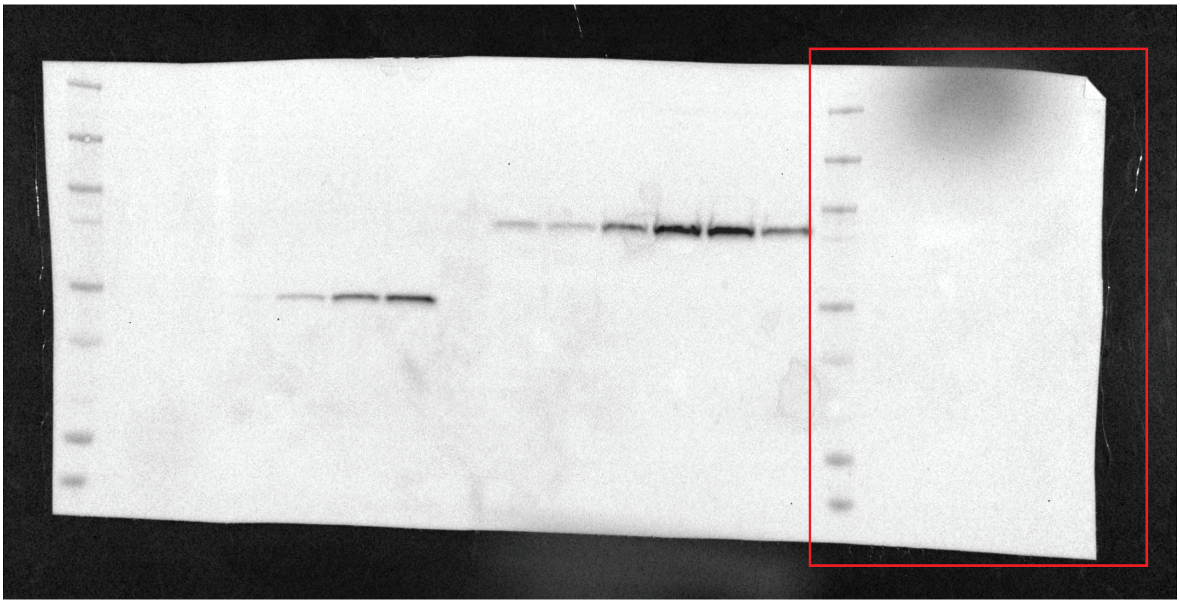

Figure 1C

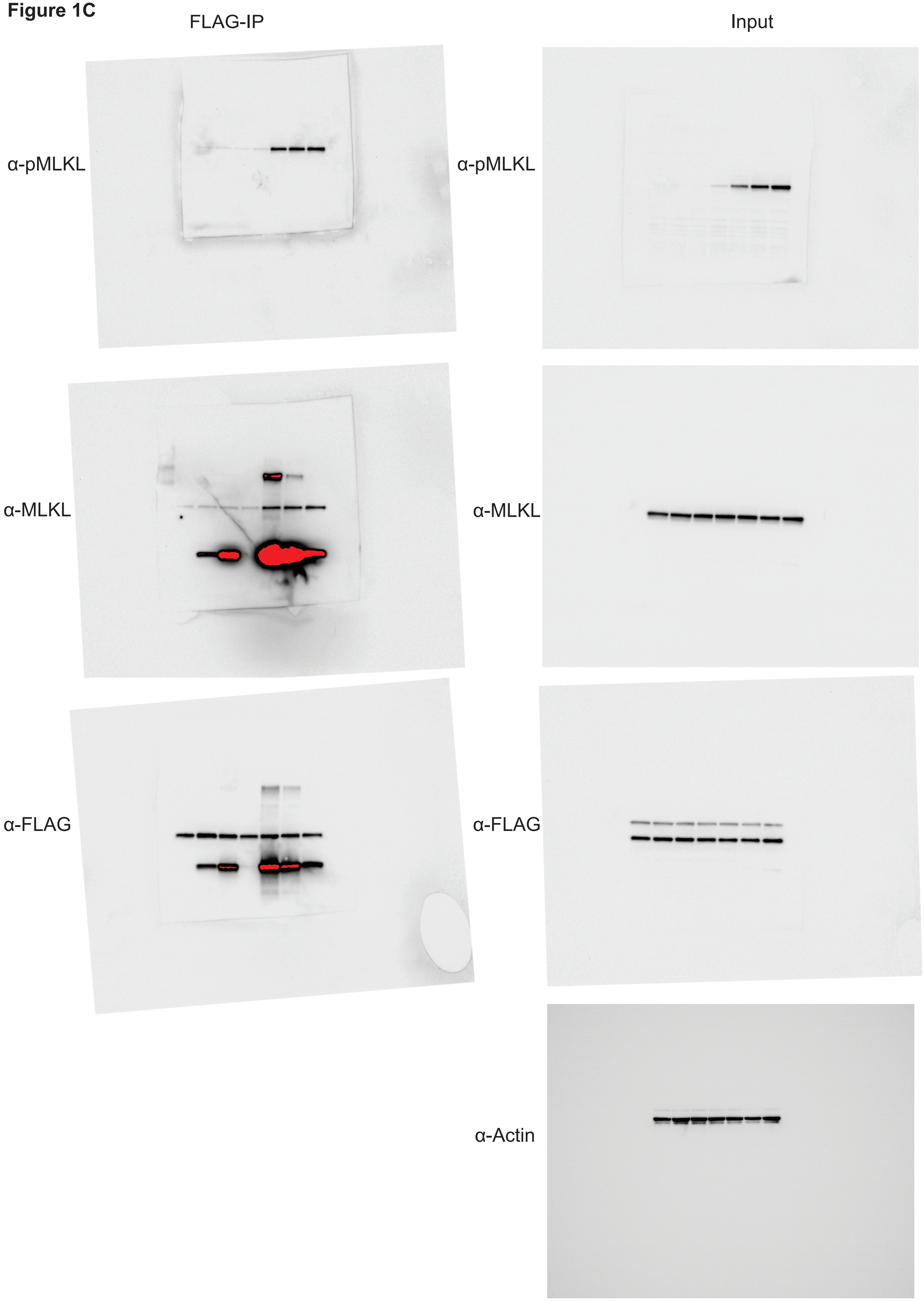

Figure 1D

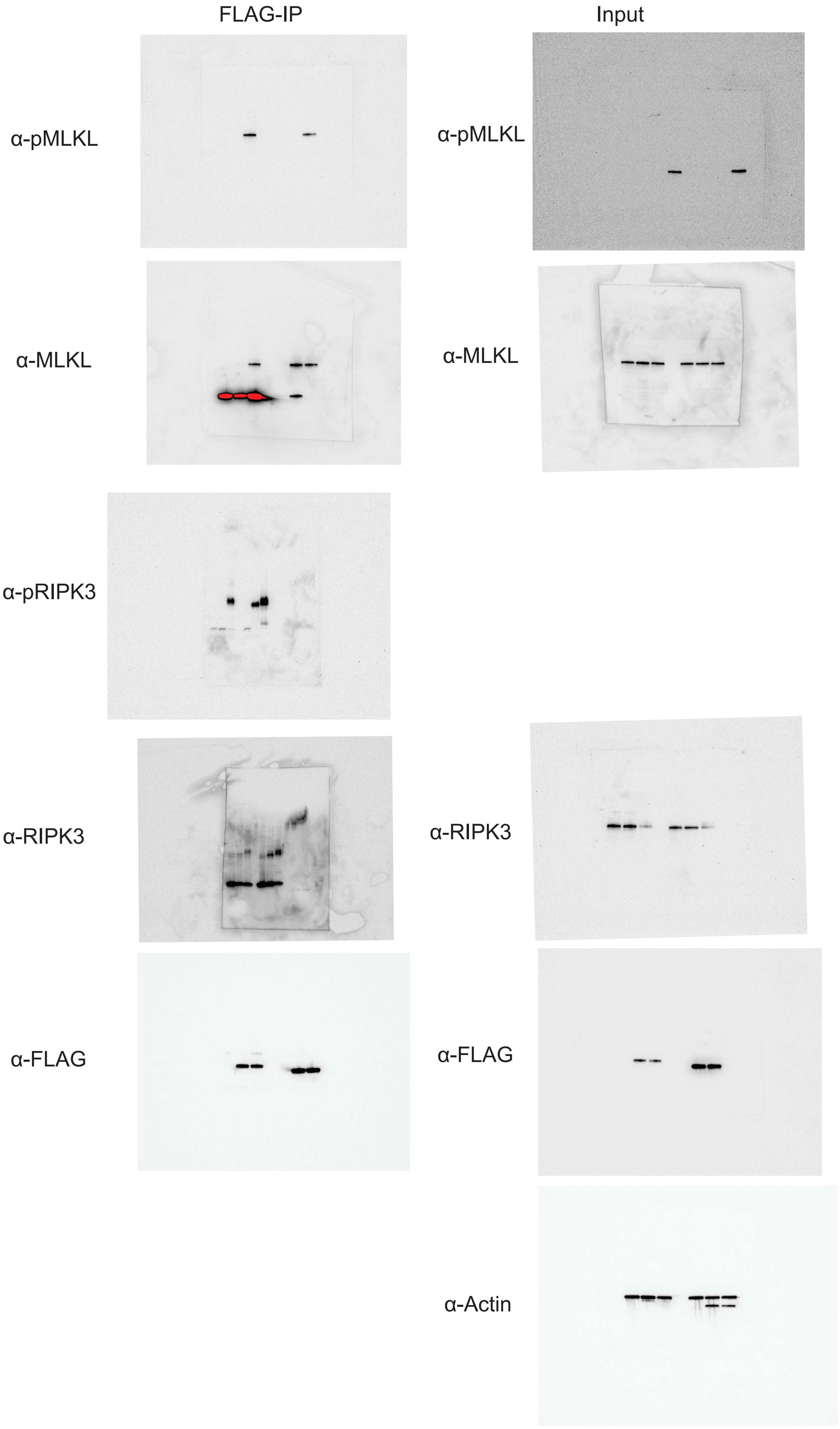
FLAG-IP
Input
α-pMLKL
α-pMLKL
α-MLKL
α-MLKL
α-pRIPK3
α-RIPK3
α-RIPK3
α-FLAG
α-FLAG
α-Actin

Figure 1E

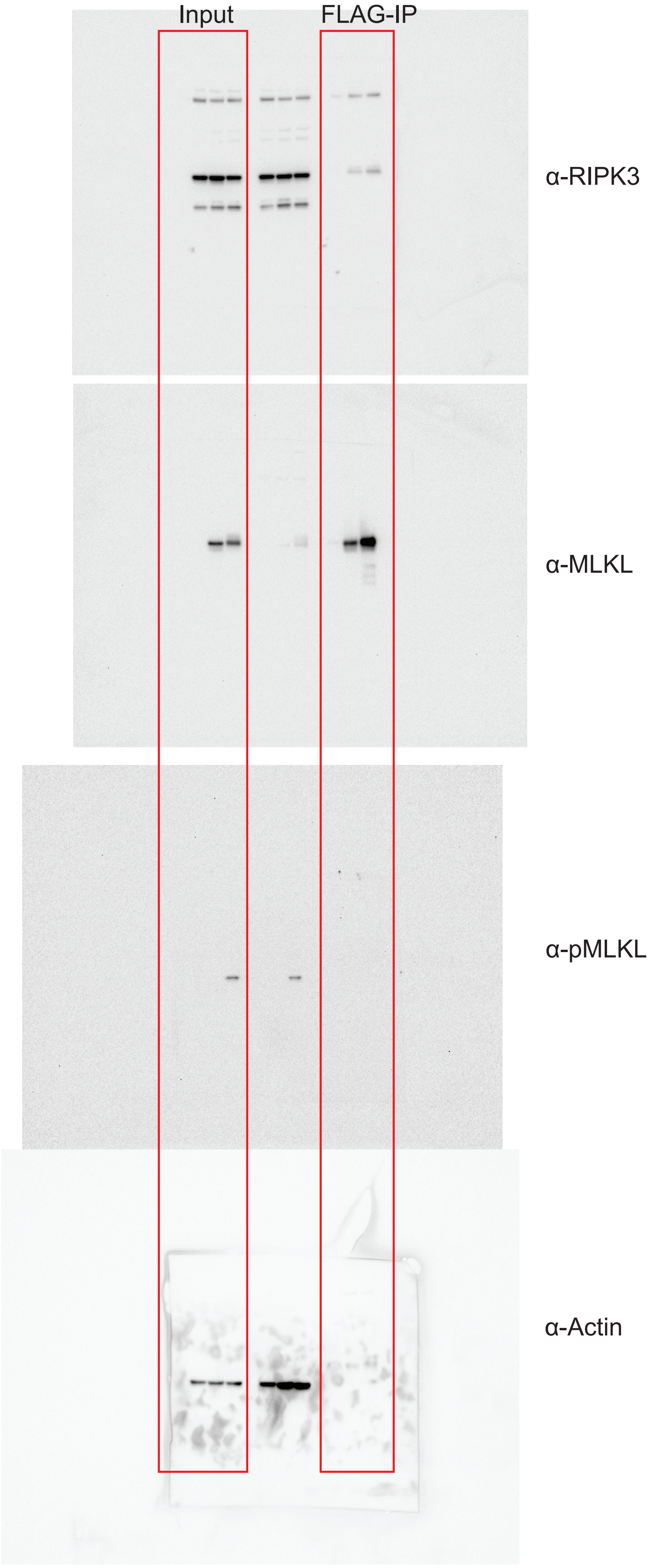

Figure 1F

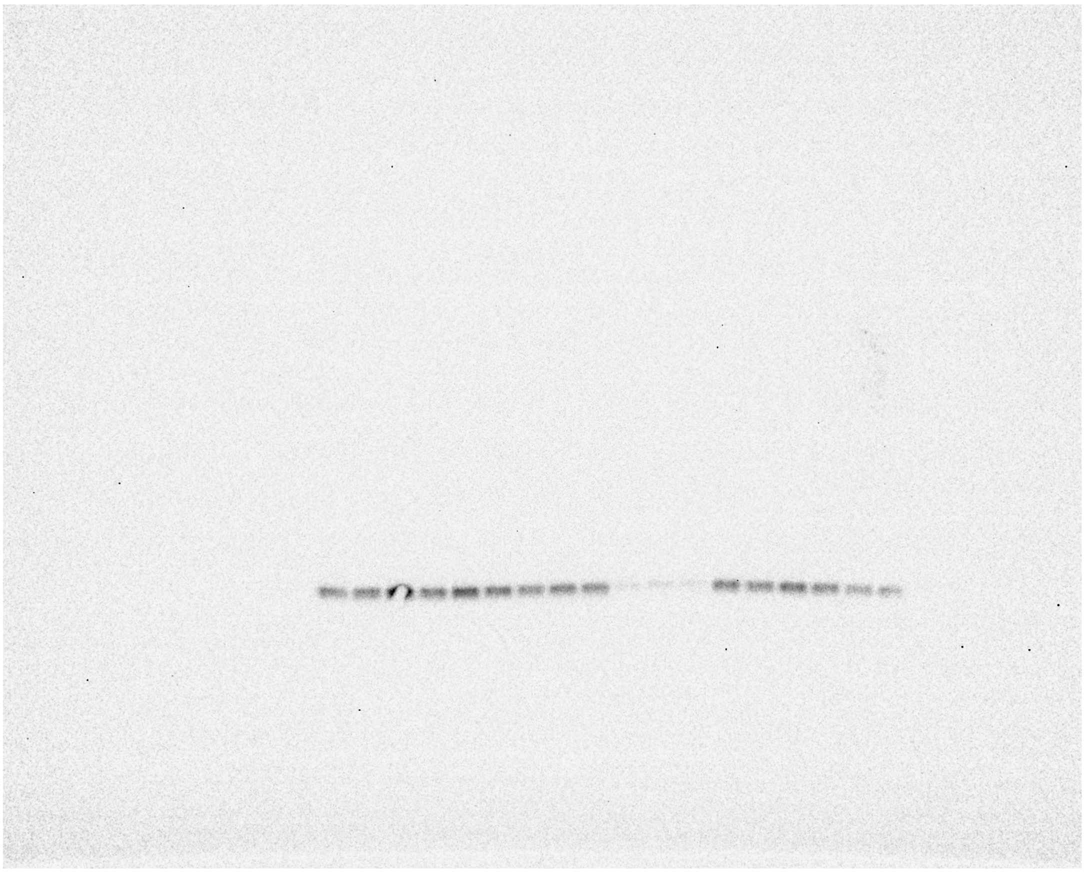

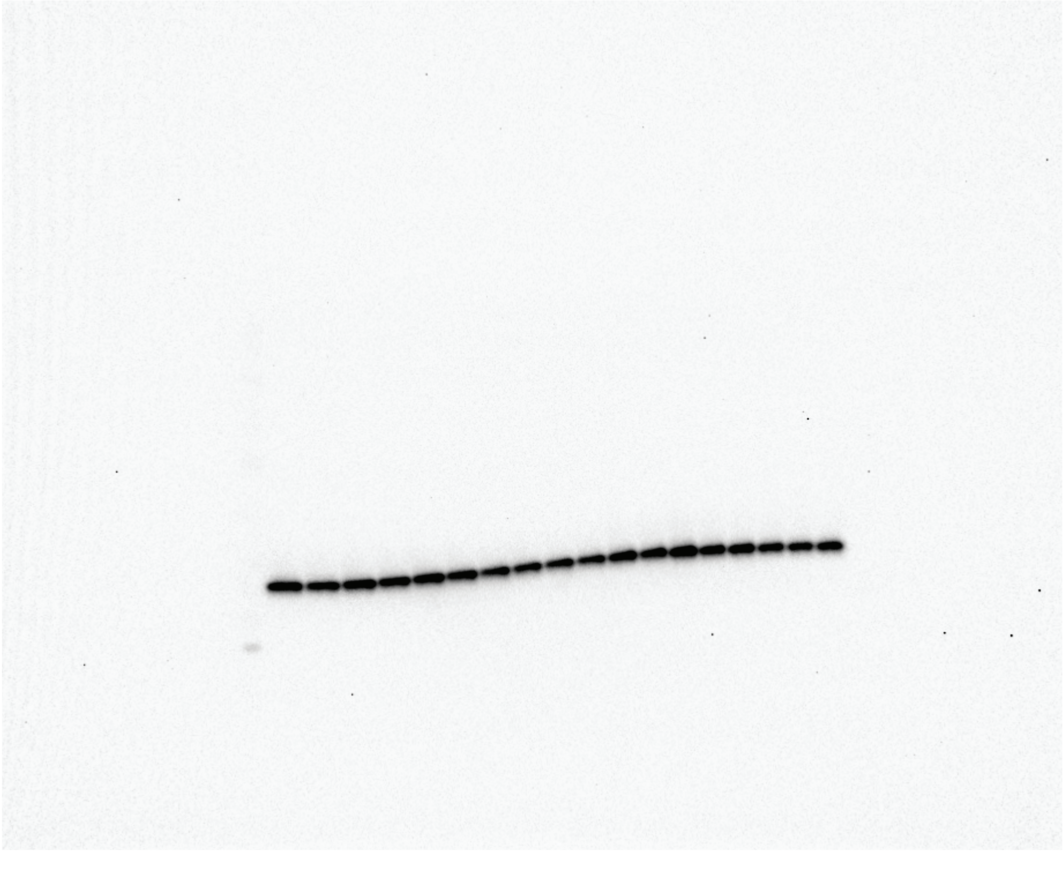

Supplementary figure 2a

α-MLKL

α-Actin

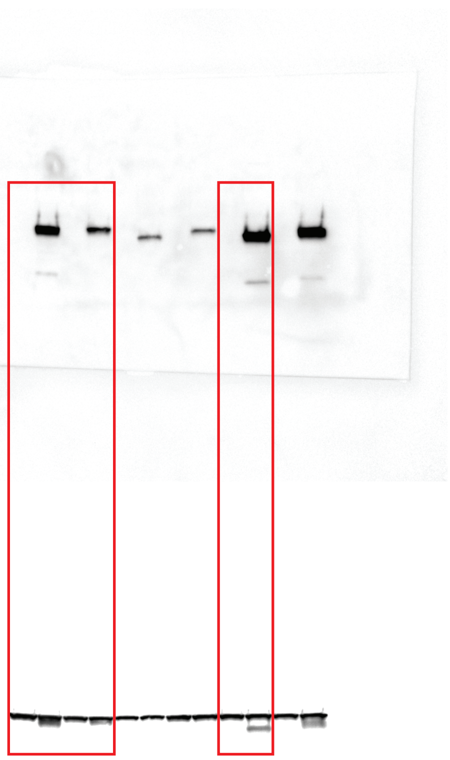

Supplementary figure 2e

FLAG-IP

α-pMLKL

α-MLKL

α-FLAG

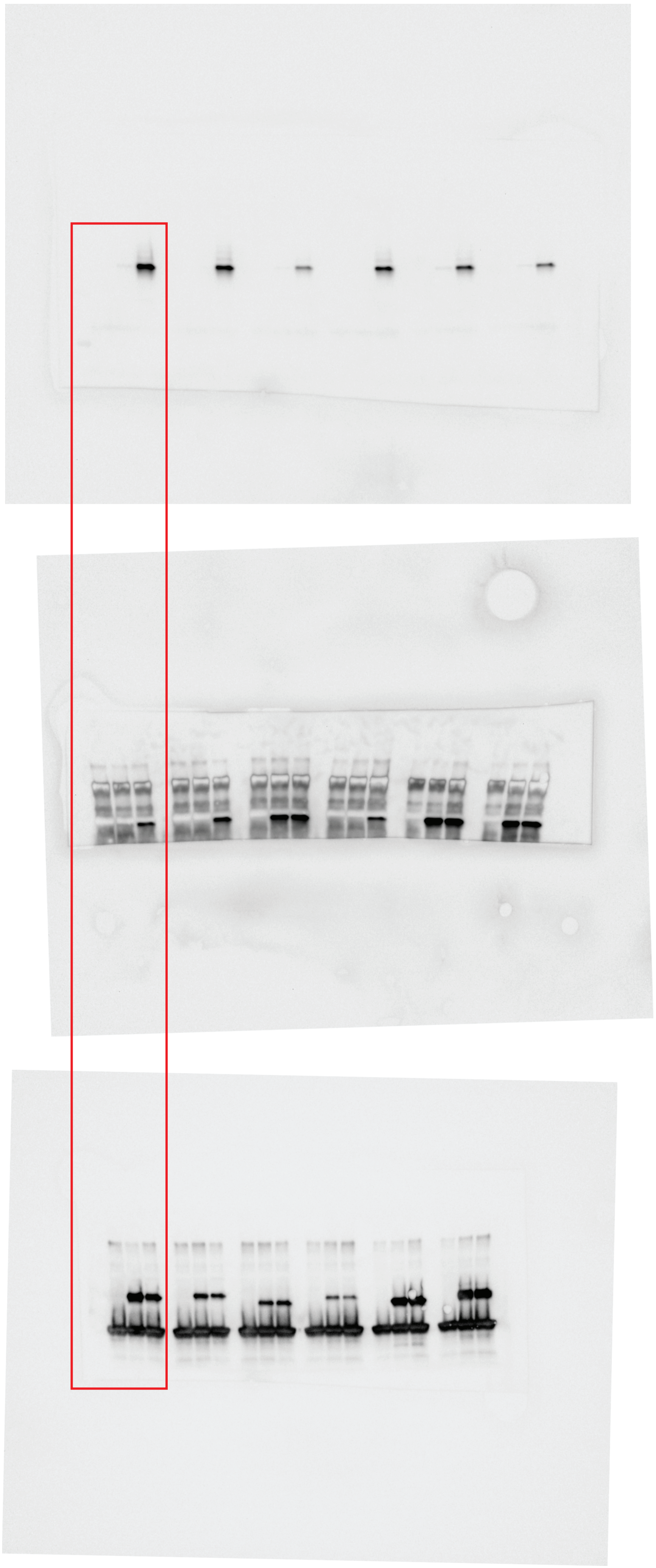

Supplementary figure 2f

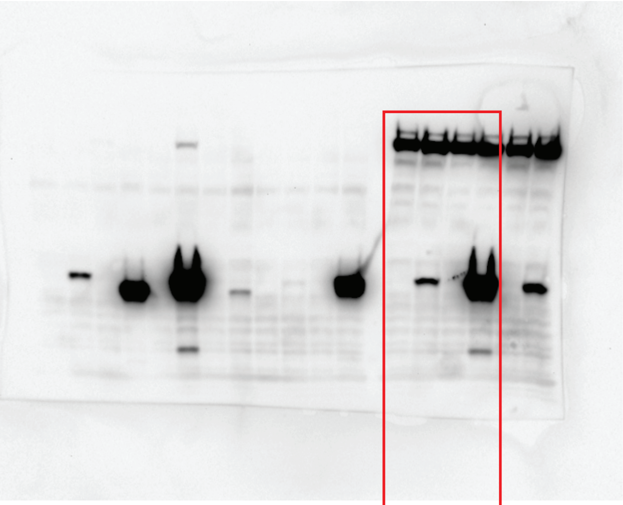

α-MLKL

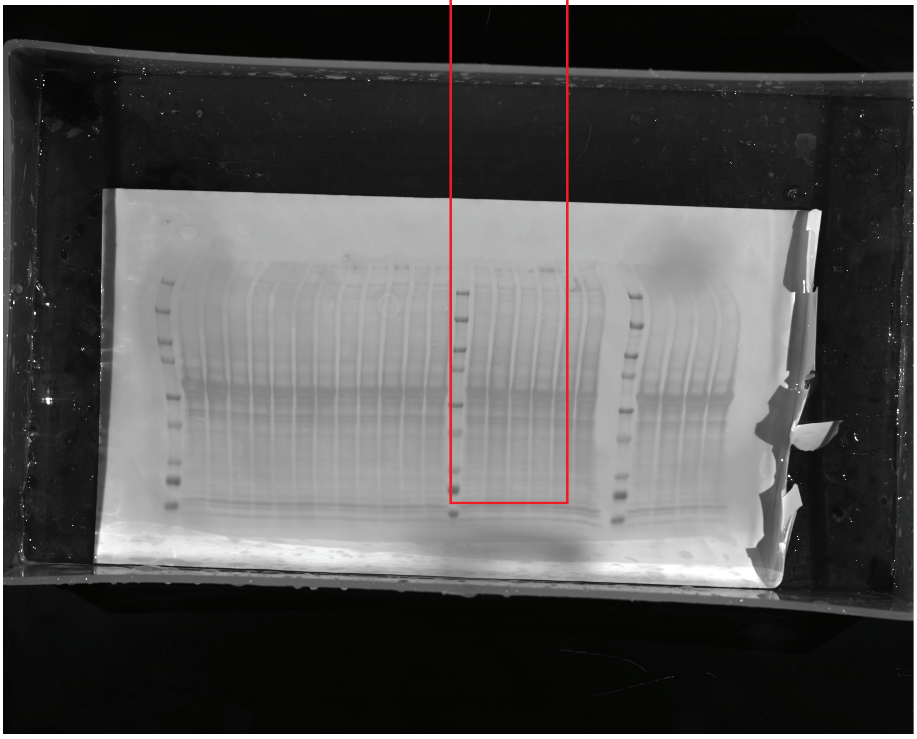

Ponceau

Supplementary figure 2g

FLAG-IP

α-MLKL

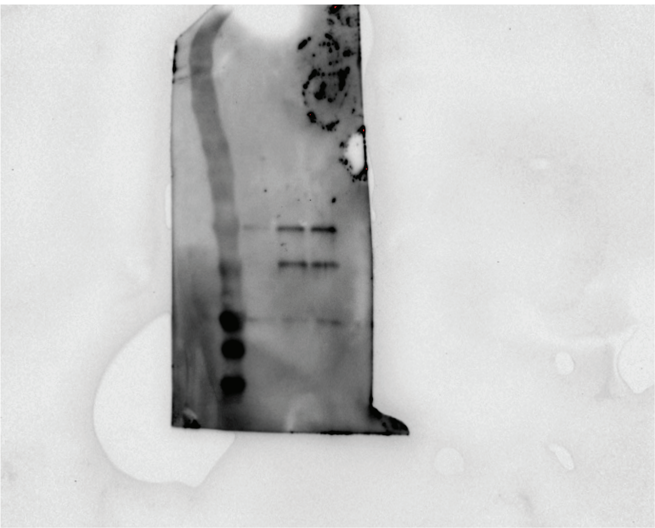

α-FLAG

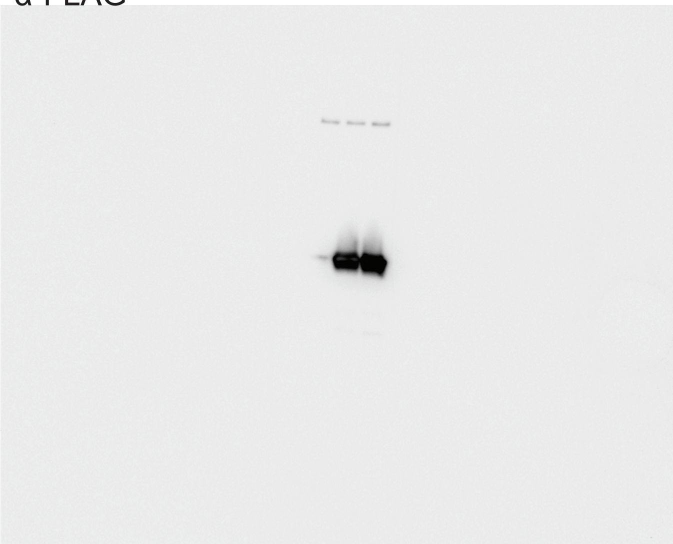

Supplementary figure 2h

α-FLAG

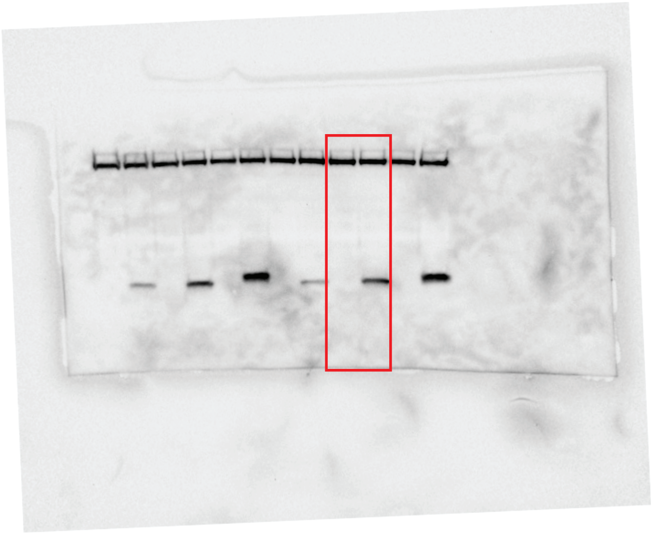

α-Actin

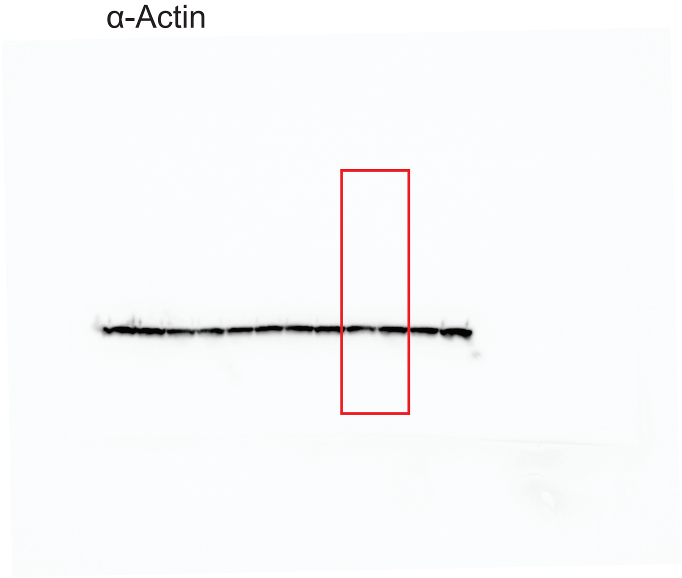

Supplementary figure 2i

FLAG-IP

α-FLAG

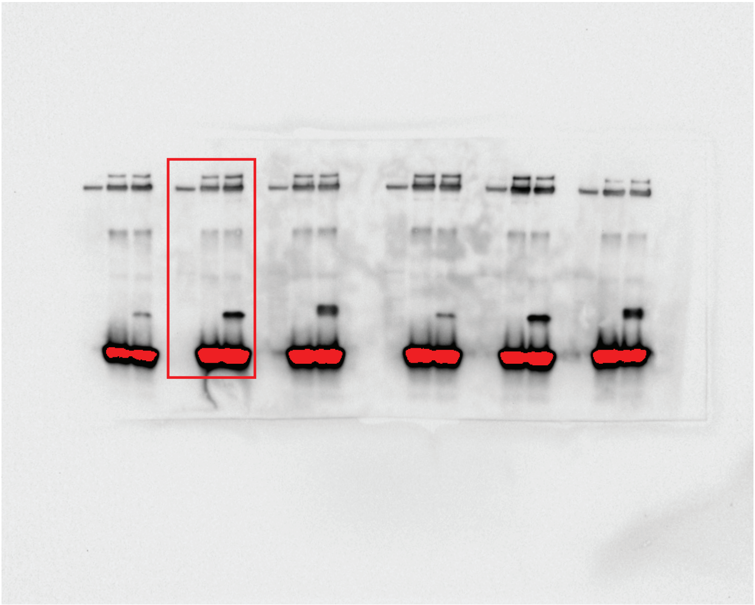

α-RIPK3

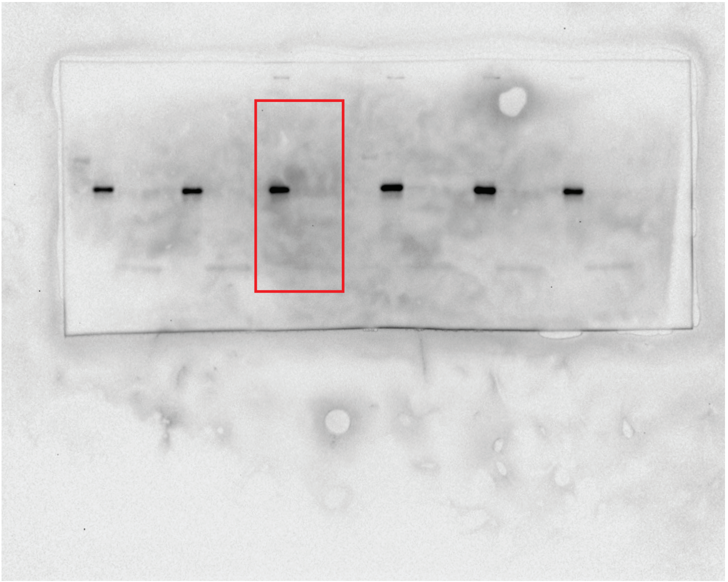

Supplementary Figure 3

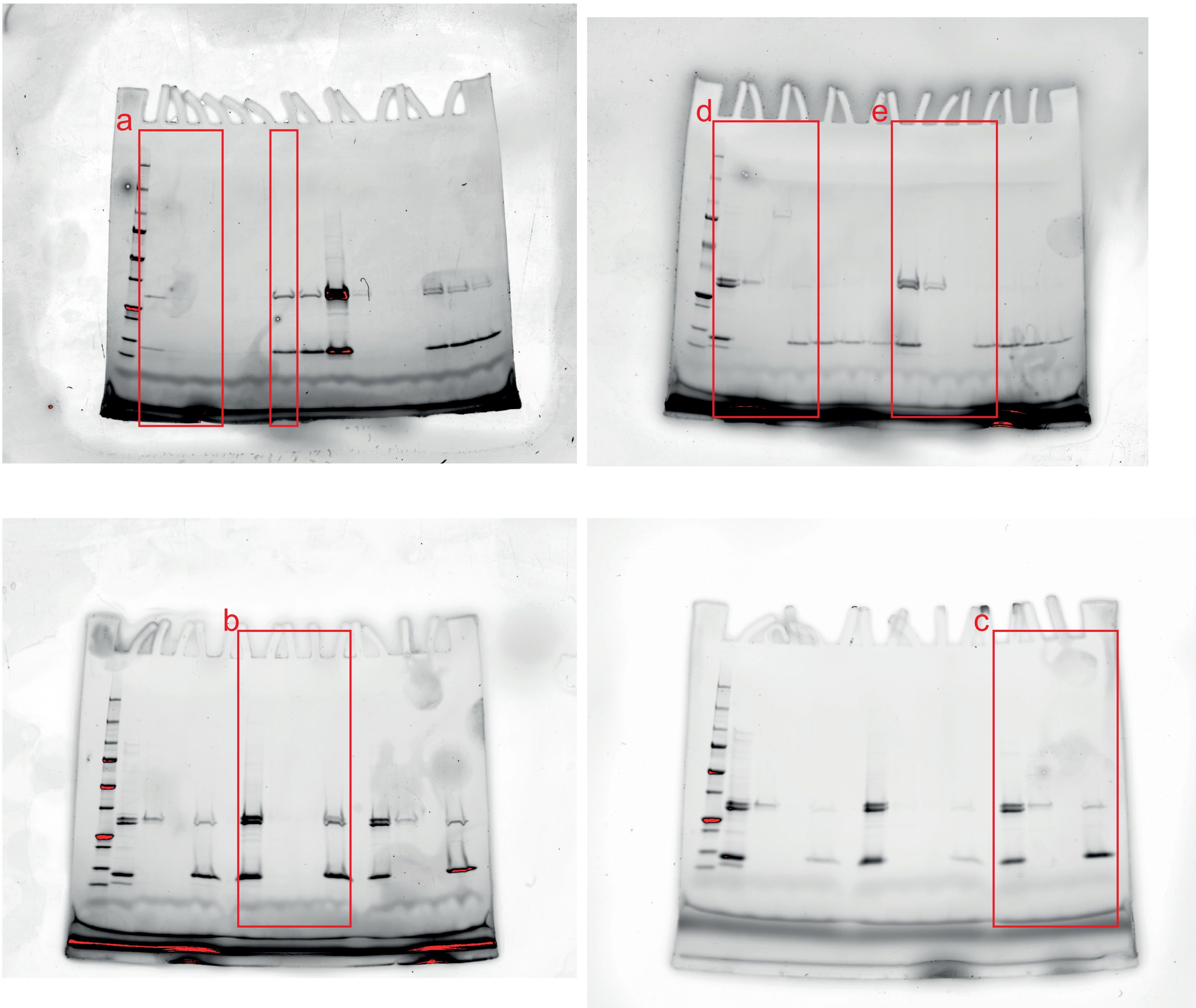

Figure 4g

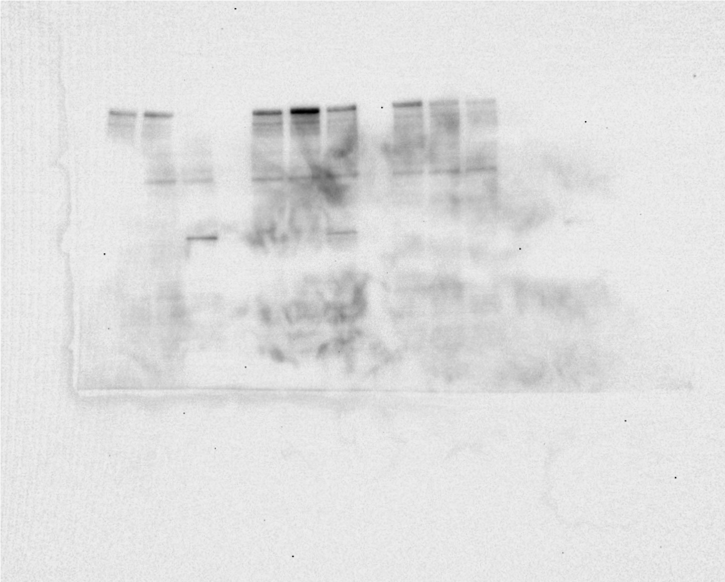

α-pMLKL

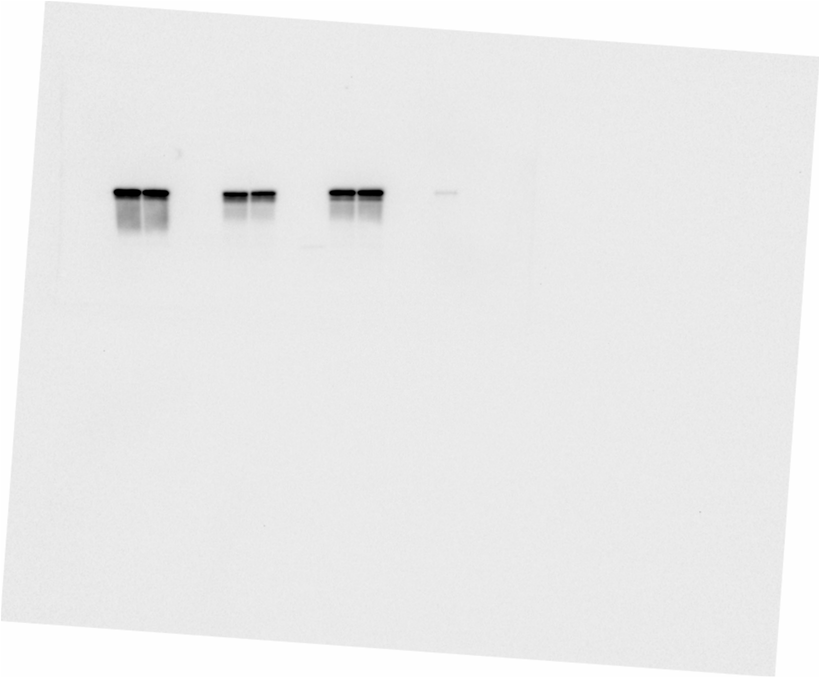

α-MLKL

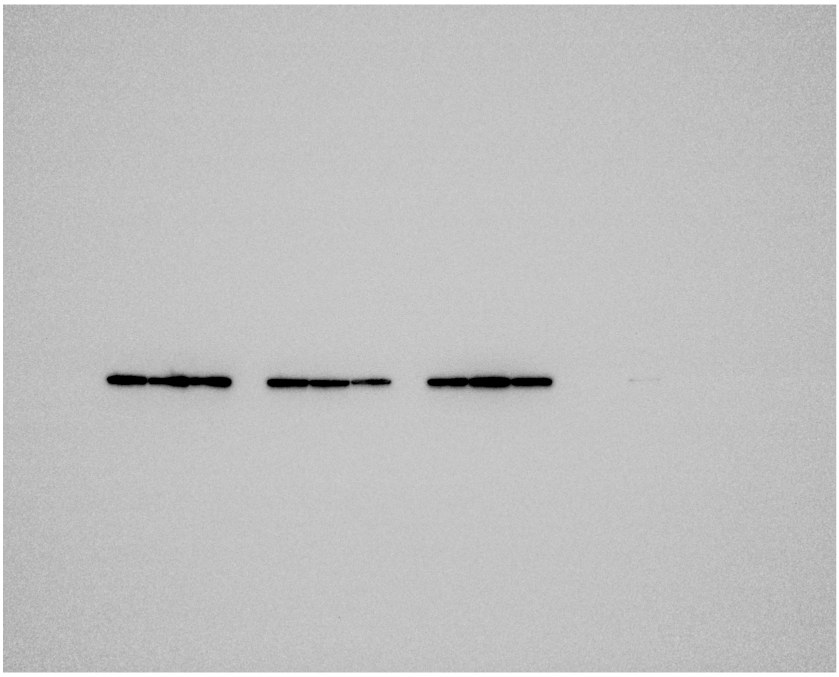

α-actin

Supplementary fig 6a

α-MLKL α-Actin

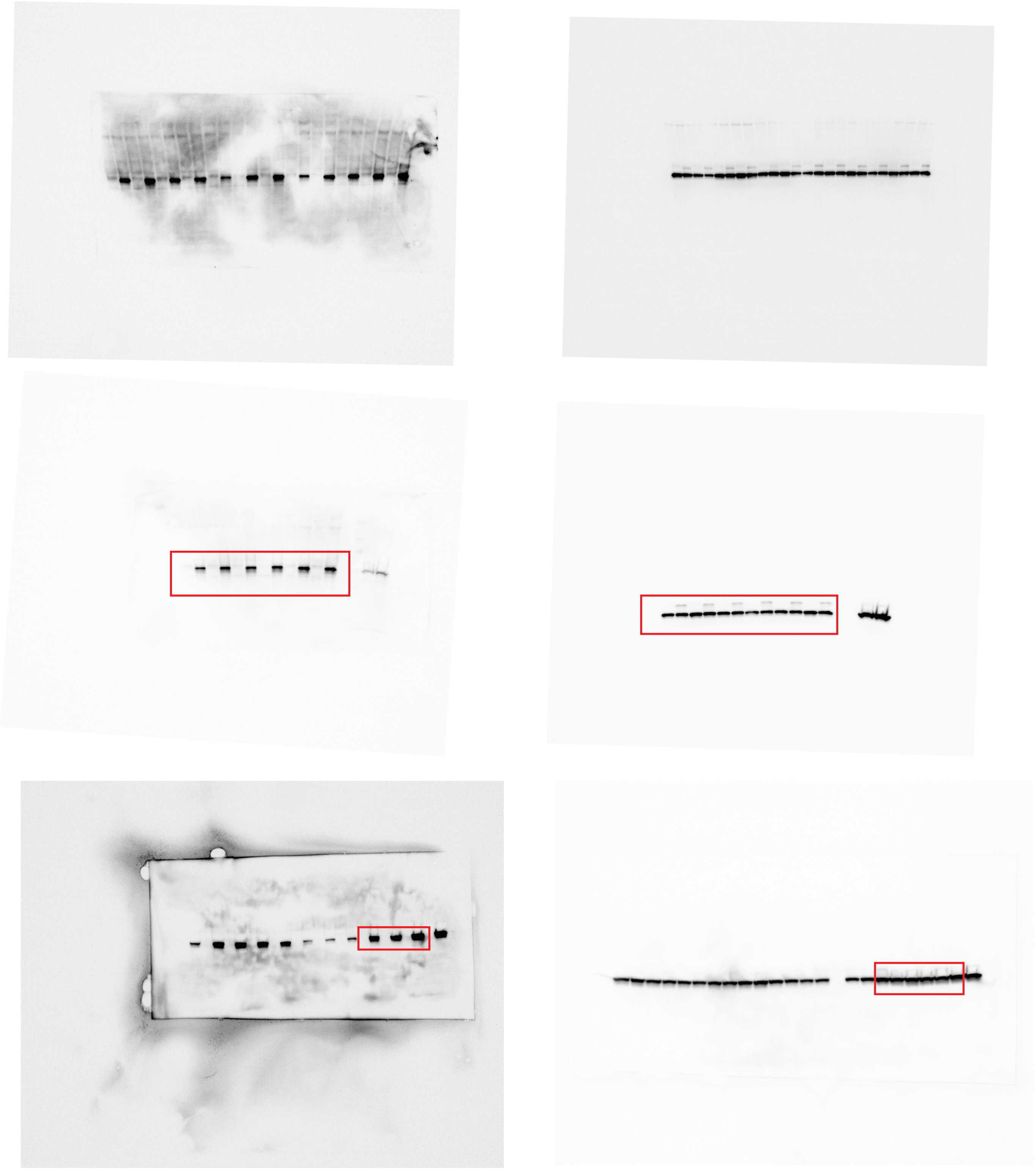

Supplementary fig 7b

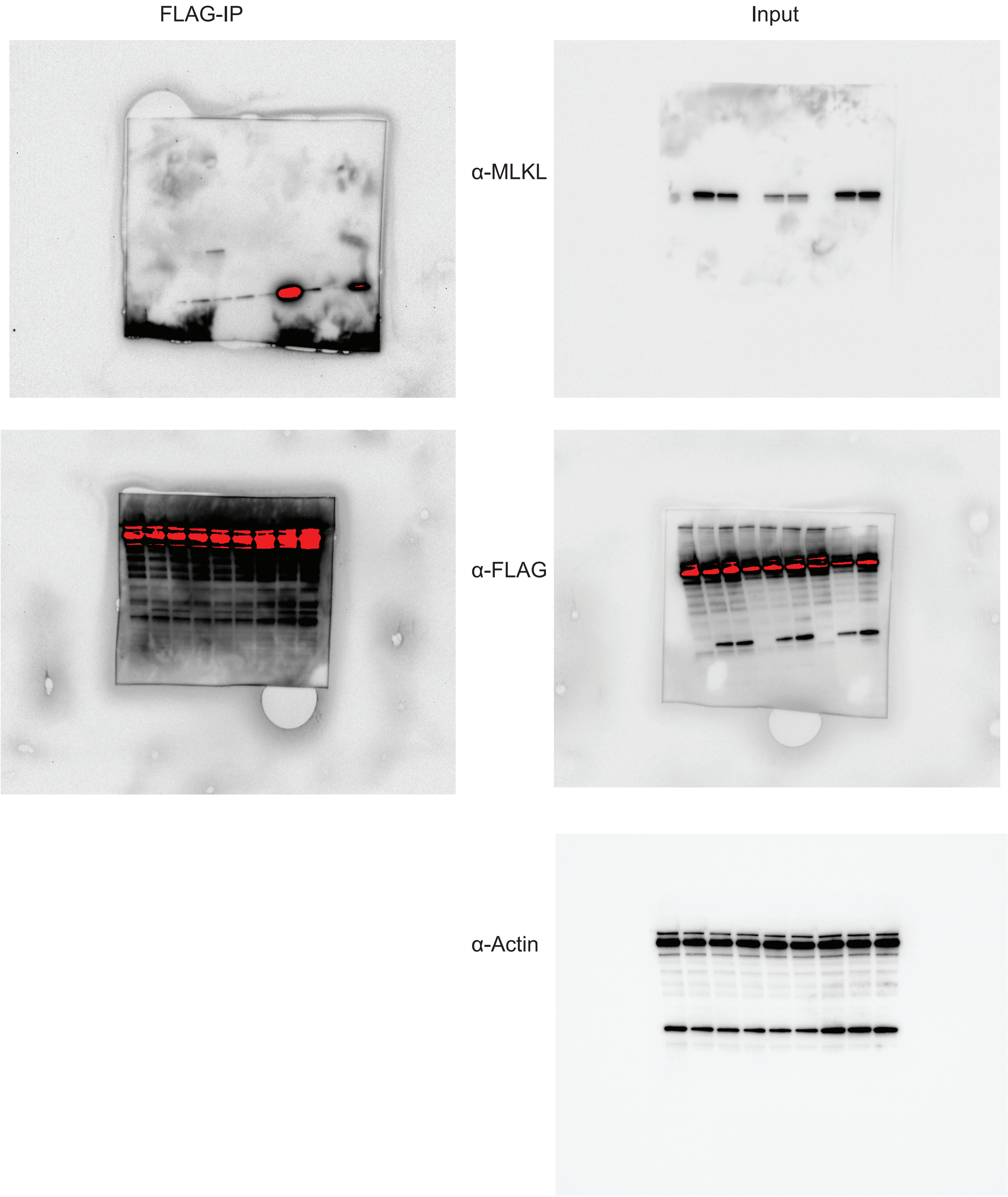

Supplement: Supplementary file 4 — Source Data [file 41467_2021_22400_MOESM4_ESM.zip › Source data/SourceData_WB.pdf]
